# The adult musculature of two pseudostomid species reveals unique patterns for flatworms (Platyhelminthes, Prolecithophora)

**DOI:** 10.1002/jmor.21039

**Published:** 2019-07-18

**Authors:** Alexandra L. Grosbusch, Philip Bertemes, Bernhard Egger

**Affiliations:** ^1^ Research Unit Evolutionary Developmental Biology Institute of Zoology, University of Innsbruck Innsbruck Austria

**Keywords:** F‐actin, phalloidin, Prolecithophora, turbellarians

## Abstract

We analyzed the adult musculature of two prolecithophoran species, *Cylindrostoma monotrochum* (von Graff, 1882) and *Monoophorum striatum* (von Graff, 1878) using a phalloidin‐rhodamine technique. As in all rhabdithophoran flatworms, the body‐wall musculature consisted of three muscle layers: on the outer side was a layer of circular muscle fibers and on the inner side was a layer of longitudinal muscle fibers; between them were two different types of diagonally orientated fibers, which is unusual for flatworms. The musculature of the pharynx consisted of a basket‐shaped grid of thin longitudinal and circular fibers. Thick anchoring muscle fibers forming a petal‐like shape connected the proximal parts of the pharynx with the body‐wall musculature. Male genital organs consisted of paired seminal vesicles, a granular vesicle, and an invaginated penis. Peculiar ring‐shaped muscles were only found in *M. striatum*, predominantly in the anterior body part. In the same species, seminal vesicles and penis only had circular musculature, while in *C. monotrochum* also longitudinal musculature was found in these organs. Female genital organs were only present in *M. striatum*, where we characterized a vagina interna, and a bursa seminalis. Transverse, crossover, and dorsoventral muscle fibers were lacking in the middle of the body and greatly varied in number and position in both species.

## 1 INTRODUCTION

Prolecithophora (=Holocoela or Cumulata) are microscopic, often drop‐shaped free‐living flatworms, that live predominantly on algae or soft sediments of marine habitats (Jondelius, Norén, & Hendelberg, 2001; Karling, 1940; Norén & Jondelius, 2002). The shape of the pharynx is variable, formed as a pharynx simplex, a pharynx plicatus, or a pharynx variabilis that point either forwards or backwards. Male and female gonads open into a common atrium genitale with a single gonopore (von Graff, 1904; Rieger & Sterrer, 1975; von Graff, 1913). However, knowledge about their morphology, phylogeny, and ecology is still scarce (Laumer & Giribet, 2017; Norén, 2004; Norén & Jondelius, 2002). Pseudostomidae von Graff, 1904, follows Plagiostomidae von Graff, 1882 as the second‐largest family within Prolecithophora and comprises 55 recognized species (Tyler, Hooge, & Bush, 2006–2016; WoRMS Editorial Board, 2019). They have a distinct brain capsule and an orogenital opening. This combined opening of mouth and genitals is on the ventral side of the posterior half of the body. Among prolecithophorans, only some pseudostomids have a bursa seminalis, either with a vagina externa (a duct debouching at the caudal end), or with a vagina interna (a duct connecting the bursa seminalis with the atrium genitale). Some species even have a bursa seminalis without any vagina (*Pseudostomum* Schmidt, 1848 and *Reisingeria* Westblad, 1955) (Karling, 1940; Westblad, 1955). Most pseudostomids have a ciliated groove anterior or posterior to the level of the brain and two pairs of eyes, but some forms have three pairs of eyes (*Reisingeria hexaoculata* Westblad, 1955), or no eyes at all (*Euxinia* von Graff, 1911) (Westblad, 1955).

*Cylindrostoma monotrochum* is a marine species. It is grayish‐brown and small (ca. 0.5–0.8 mm long). It is lacking a vagina and a bursa seminalis, but has paired germaries, one on either side of its conical and remarkably broad pharynx (von Graff, 1882; Karling, 1962; von Graff, 1913; Westblad, 1955). *Monoophorum striatum* is a marine representative as well. It is opaque with red, longitudinal stripes and has a length up to 1.5 mm. It has an unpaired dorsal germarium and a bursa seminalis with a characteristic vagina interna debouching into the common atrium. Inside the granular vesicle, there is a strikingly long penis (Böhmig, 1890; von Graff, 1882; von Graff, 1913; Westblad, 1955).

Body‐wall musculature is key to maintain the outer body shape of flatworms. Characters of the musculature can be used to study taxonomic relationships of Platyhelminthes Minot, 1876 (Hooge, 2001; Rieger, Tyler, Smith III, & Rieger, 1991; Tyler & Hooge, 2004). In general, the musculature of flatworms is divided into body‐wall musculature and inner body musculature. Catenulids show a simple network of body‐wall muscle fibers consisting of outer circular and inner longitudinal muscle layers. In Rhabditophora Ehlers, 1985, the body‐wall musculature consists of three different types of layers. An outer layer of circular fibers enwraps the whole body, an inner layer of longitudinal fibers stretches over the entire length of the body, and a layer of diagonal fibers is generally situated between the other two layers (Ehlers, 1985; Hooge, 2001; Rieger et al., 1991; Tyler & Hooge, 2004). Sometimes, several more layers of circular, diagonal, and longitudinal muscles are found, for example, in polyclads (Prudhoe, 1985).

The inner body musculature mainly consists of the musculature of the genital complex and the pharynx, as well as of the associated fine muscle fibers (Hooge, 2001; Hooge & Tyler, 1999; Rieger et al., 1991). Musculature of the gut is only described in some flatworm orders (von Graff, 1882; Rieger et al., 1991). Only few studies describe the musculature of the genital organs. Generally, male genital organs have a reinforced musculature built of one or more layers of circular and longitudinal muscle fibers, and female genital organs have either weak or no musculature (Böhmig, 1890; Girstmair, Schnegg, Telford, & Egger, 2014; von Graff, 1882; Hooge & Tyler, 1999; Karling, 1940). Dorsoventral musculature is well developed only in larger free‐living flatworms, namely in polyclads and triclads. Most other groups have scarce dorsoventral muscles, and only in some parts of the body (Ehlers, 1985; Rieger et al., 1991). In several flatworm taxa, muscle fibers enclose or traverse the brain (Girstmair et al., 2014; Orii, Ito, & Watanabe, 2002; Rieger, Salvenmoser, Legniti, & Tyler, 1994).

In this study, we compare the F‐actin muscle patterns of two pseudostomids, *C. monotrochum* and *M. striatum*, using a phalloidin‐rhodamine staining method on whole‐mount adults (Rieger et al., 1994; Rieger & Salvenmoser, 1991).

## 2 MATERIAL AND METHODS

### 2.1 Animals

Specimens of *C. monotrochum* and *M. striatum* were collected from brown algae in the port of Punat, Krk, Croatia (45°01′23″N 14°37′41″E) in March 2016, October 2016, 2017 and in May 2018. The worms were extracted from the algae using a 1:1 7.14% MgCl_2_ × 6H_2_O and artificial sea water (ASW) solution, and identified as the two species by histological sections (see below). Animals were maintained in petri dishes with ASW in a climate chamber, at 18°C with 60% humidity and a 14:10 hours day‐night cycle.

### 2.2 Phalloidin‐rhodamine technique

For whole‐mount stainings, animals were relaxed in a 1:1 7.14% MgCl_2_ × 6H_2_O and ASW solution for 15–20 min, fixed in 4% formaldehyde (made from paraformaldehyde) in 1× phosphate buffered saline (PBS) for 1 hr at room temperature (RT) and washed with PBS‐T_x_ (PBS with 0.1% Triton X‐100, Sigma‐Aldrich) for 1 hr. Specimens were blocked for 1 hr at RT with BSA‐T_x_ (PBS‐T_x_ with 1% bovine serum albumin, Carl Roth, Germany) and incubated overnight at 4°C in a rabbit anti‐5HT antibody (Sigma‐Aldrich) diluted 1:2,000 in BSA‐Tx (data from antibody staining not shown here). After being washed with PBS‐T_x_ for 2 days, specimens were incubated in tetramethylrhodamine‐conjugated phalloidin (P1951; Sigma‐Aldrich) and the secondary fluorescein isothiocyanate‐conjugated (FITC) swine anti‐rabbit antibody (Dako, Denmark), both diluted 1:250 in BSA‐T_x_ for 1 hr at room temperature in darkness. Subsequently, specimens were rinsed with PBS‐T_x_ for 3 days at RT and three nights at 4°C in darkness. Finally, they were mounted in Vectashield (Vector Laboratories). In total, 20 adult *M. striatum* and five adult *C. monotrochum* were stained.

### 2.3 Histological sections and stainings

Three adult animals of each species were relaxed and fixed as described above. Then they were rinsed with distilled water for 5 min and dehydrated in a graded series of ethanol. Subsequently, specimens were infiltrated with a 1:1 mixture of ethanol and Technovit 7100 (Kulzer Technik, Germany) overnight. Then, they were transferred to 100% Technovit 7100 overnight. Finally, specimens were embedded in Technovit resin overnight at RT and stuck to a wooden block with Technovit 3040.

Semi‐thin sections at 3 μm thickness were made with a microtome (Reichert‐Jung Autocut 2040) and stained using a standard hematoxylin‐eosin (H.E.) protocol (Harris hematoxylin and 5% eosin‐Y in ethanol).

### 2.4 Microscopy and visualization

Live squeeze preparations and H.E. stained sections were observed using a Leica DM 5000 B microscope and photographs were taken with a Leica DFC 490 camera. Confocal stacks were generated using a Leica TCS SP5 II confocal microscope and processed with the open‐source program Fiji v. 1.52j (Schindelin et al., 2012). Depth‐color‐coded images were done by using the look‐up tables “Ice” and “Spectrum” included in Fiji. Picture editing and drawings were done with the open‐source programs GIMP up to v. 2.10 (http://www.gimp.org) and Inkscape v. 0.92 (https://inkscape.org).

## 3 RESULTS

### 3.1 Body‐wall musculature

#### 3.1.1 General characteristics of the body‐wall musculature

**Figure 1:**
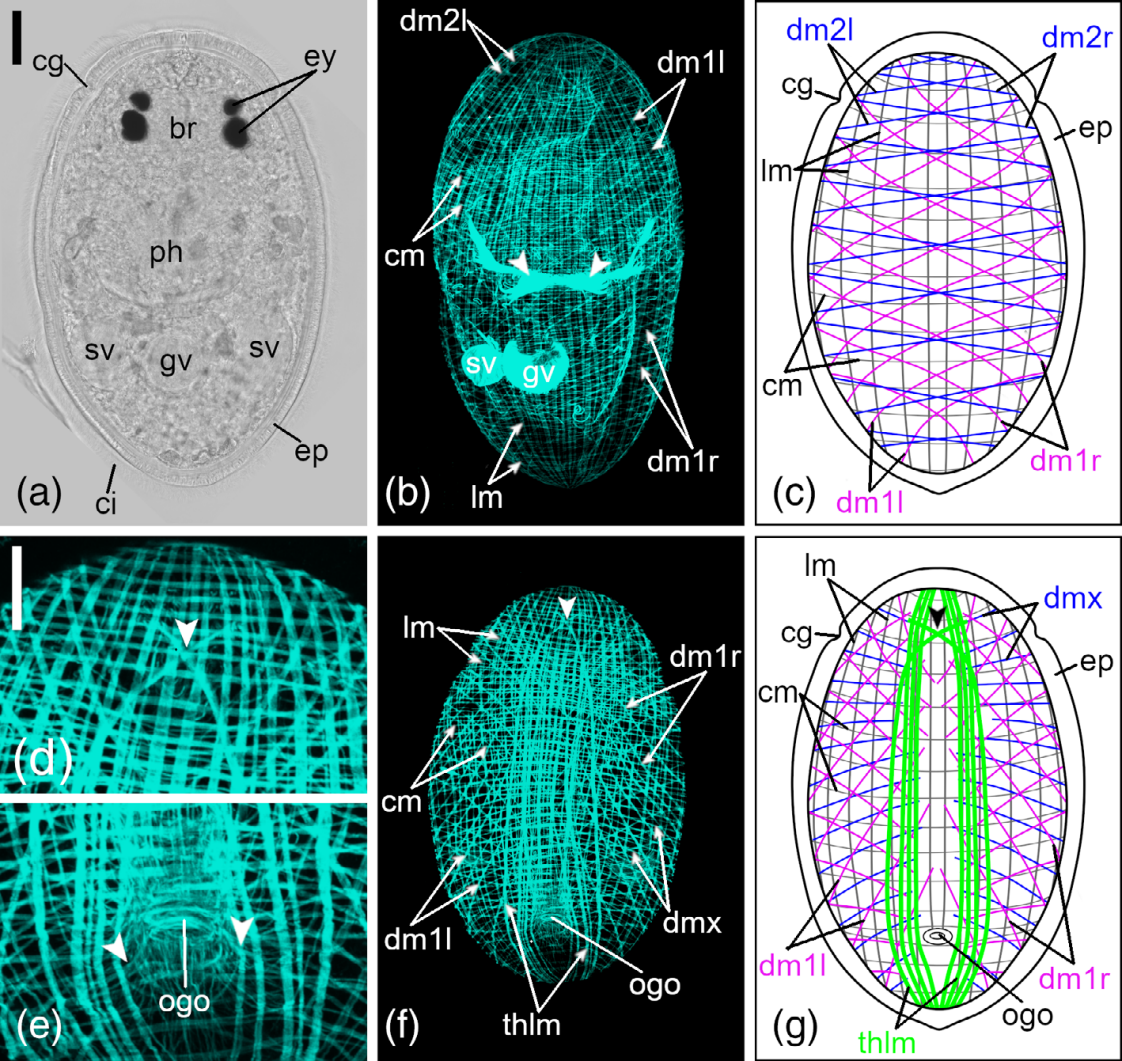
Body‐wall musculature of *Monoophorum striatum*. (a) Dorsal view of a fixed adult specimen. (b) Projection and (c) schematic drawing of the dorsal body‐wall musculature. Arrowheads in (b) show anchoring muscles of the muscularis externa of the pharynx. (d–f) Ventral projections: (d) close‐up of the anterior tip of the body; (e) close‐up of the orogenital opening; arrowheads show diagonal muscle fibers, bending toward the orogenital opening. (f) Projection and (g) schematic drawing of the ventral body‐wall musculature. Arrowheads in (d,f,g) show the outermost pair of the thickened longitudinal muscle bands which cross each other at the body midline. Anterior is up for all animals. *Scale bars*: (a,b,f) 50 μm and (d and e) 25 μm. br, brain; ci, cilia; cg, ciliary groove; cm, circular muscle rings; dm1, diagonal muscle fibers of the first type running from anteriad right to posteriad left (dm1l) or from anteriad left to posteriad right (dm1r); dm2, diagonal muscle fibers of the second type on the dorsal side running from anteriad right to posteriad left (dm2l) or from anteriad left to posteriad right (dm2r); dmx, diagonal muscle fibers on the ventral side which cannot be clearly attributed to either dm2l or dm2r; ep, epidermis; ey, two pairs of eyes; gv, granular vesicle; lm, longitudinal muscle fibers; ph, pharynx; sv, paired seminal vesicles; thlm, thickened longitudinal muscles; ogo, orogenital opening

**Figure 2:**
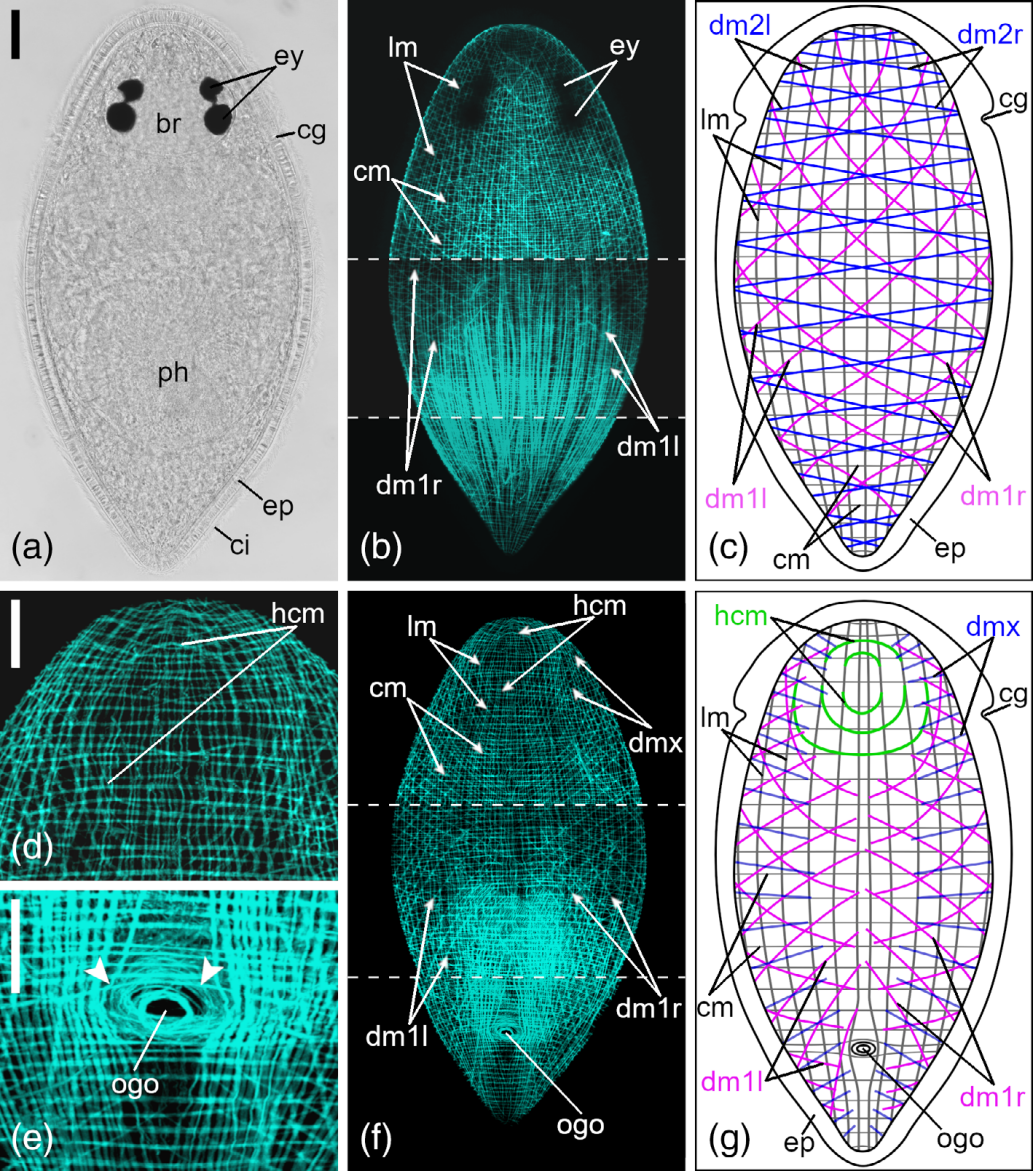
Body‐wall musculature of *Cylindrostoma monotrochum*. (a) Dorsal view of a fixed adult specimen. (b) Projection and (c) schematic drawing of the dorsal body‐wall musculature. (d–f) Ventral projections: (d) close‐up of the anterior tip of the body; (e) close‐up of the orogenital opening; arrowheads show circular muscle fibers, bending toward the orogenital opening. (f) Projection and (g) schematic drawing of the ventral body‐wall musculature. Dashed lines in (f) indicate borders between three separate confocal stacks that were subjected to different brightness levels. Anterior is up for all animals. *Scale bars*: (a,b,f) 50 μm and (d,e) 25 μm. br, brain; ci, cilia; cg, ciliary groove; cm, circular muscle rings; dm1, diagonal muscle fibers of the first type running from anteriad right to posteriad left (dm1l) or from anteriad left to posteriad right (dm1r); dm2, diagonal muscle fibers of the second type on the dorsal side running from anteriad right to posteriad left (dm2l) or from anteriad left to posteriad right (dm2r); dmx, diagonal muscle fibers on the ventral side which cannot be clearly attributed to either dm2l or dm2r; ep, epidermis; ey, two pairs of eyes; hcm, half‐circle muscle fibers; lm, longitudinal muscle fibers; ph, pharynx; ogo, orogenital opening

The general pattern of the body‐wall musculature of both species consists of an outer layer of circular muscle rings (cm), an inner layer of longitudinal muscle fibers (lm) and a layer of diagonal muscle fibers (dm) in between (Figures 1b,c,f,g and 2b,c,f,g). The rings are evenly spaced and encircle the full circumference of the body. On the ventral side at the posterior body half, cm slightly bend around the orogenital opening (Figures 1e and 2e).

On the dorsal side, sets of two different types of dm (dm1 and dm2) extend over the total body length (Figures 1b,c,f,g and 2b,c,f,g). Dm1 and dm2 are each composed of two opposing strands, running either from anteriad left to posteriad right (dm1r/dm2r) or from anteriad right to posteriad left (dm1l/dm2l). The paired opposing strands in these sets cross over at the body midline (Figures 1c and 2c). On the ventral side, also two different types of dm (dm1 and dmx) are present, but only dm1 are composed of opposing strands (dm1r/dm1l) (Figures 1g and 2g). The dorsal dm1 and dm2 extend across the entire dorsal side, but ventral dm1 and dmx reach only to the body midline (Figures 1g and 2g). According to lateral views of some individuals, ventral dm1 are continuous with dorsal dm1, but dmx cannot be clearly attributed to either dm2l or dm2r. Adjoining fibers of both types of dm are more or less evenly spaced from each other and interspaces were 2 to 3 times as large as those of lm and cm. At the anterior quarter of the body, the angle of dm1 to the longitudinal body axis is smaller than more posteriorly (Figures 1b,c,f,g and 2b,c,f,g). Dm2 have a uniform, but greater angle to the longitudinal body axis all over the body (Figures 1b,c,f,g and 2b,c, f,g). On the ventral side of the posterior quarter of the body, dm2 run toward the orogenital opening (Figures 1e and 2e).

Lm run perpendicular to the cm along the entire body length (Figures 1b,c,f,g and 2b,c,f,g). Most lm bend toward the center of the tip of the body and end at the anterior‐most and posterior‐most tip (data not shown). Some muscle fibers branch off from their band and either fuse to an adjacent muscle band or appear to end loosely (data not shown).

#### 3.1.2 Differences between body‐wall musculature of *M. striatum* and *C. monotrochum*

While the outer layer of the body‐wall musculature in *M. striatum* is built of approximately 60 unevenly spaced cm (about 40 circular muscles per millimeter), in *C. monotrochum* the outer layer is built of approximately 105 evenly spaced cm (161 circular muscles per millimeter; Figures 1b,c,f,g and 2b,c,f,g). Approximately 45 lm (195 lm per mm), which are unevenly spaced, build the third layer of the body‐wall of *M. striatum* (Figure 1b,c,f,g). In *C. monotrochum*, lm are evenly spaced, and they occur in different numbers on the ventral side than on the dorsal side. Approximately 85 lm (303 lm per mm) are on the dorsal side and about 65 lm (232 lm per mm) on the ventral side (Figure 2b,c,f,g). Only in *M. striatum*, there is a lack of lm in the area between the sphincter muscle of the orogenital opening and the posterior‐most tip of the ventral side of the body, as well as an additional set of 10 thickened lm beside the ventral body midline (Figure 1e–g). The outermost pair of the thickened lm cross each other at the body midline in the anterior part of the body and then bend dorsally toward a pair of dorsoventral muscles anterior to the brain (Figure 1d). On the anterior part of the ventral side, some muscle fibers branch off from lm and run toward the middle, where they fuse and form half circles, a feature found only in *C. monotrochum* (Figure 2d,f,g).

### 3.2 Inner‐body muscles

#### 3.2.1 General characteristics of the pharyngeal musculature

**Figure 3:**
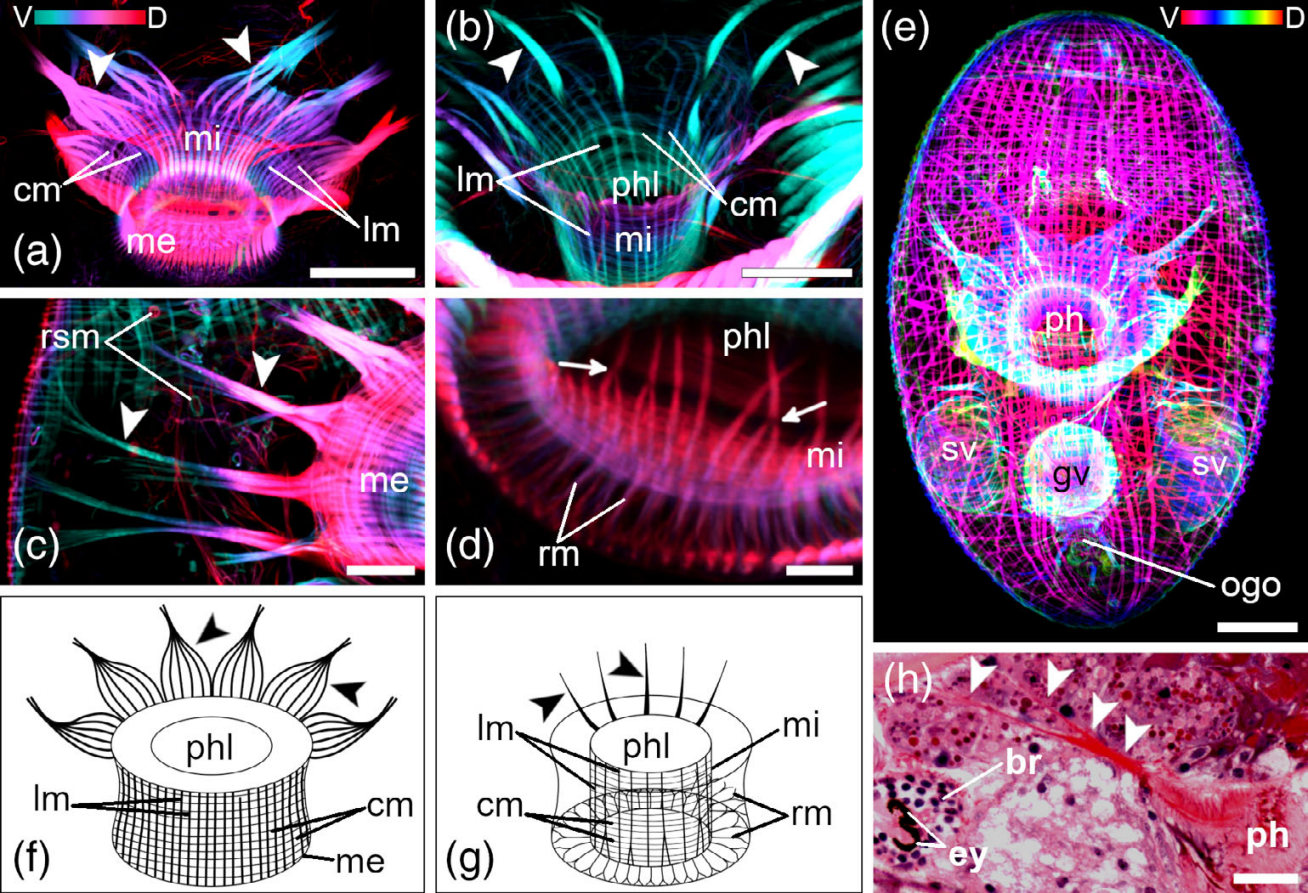
Pharyngeal musculature of *Monoophorum striatum*. (a–d) Depth‐color‐coded central projections, blue is more ventral, red more dorsal. White structures originate from an accumulation of individual hues from corresponding pixels across each plane of the confocal stack during image processing. (a) Overview of the whole pharynx. (b) Detail of the inner pharynx musculature. (c) Connection of the pharynx with the body‐wall. (d) Detail of the distal pharynx musculature connecting muscularis interna and externa with radial muscle fibers. Arrows show branching points of lm of the muscularis interna. (e) Depth‐color‐coded projection of a whole animal; red is more ventral, orange more dorsal. (f,g) Schematic drawings of the muscularis externa (f) and interna (g). Arrowheads in (a–c, f‐h) point at the longitudinal muscle fibers (anchoring muscles) radiating out of the muscularis externa (a,c,f), and in (b,g) of the muscularis interna. (h) Histological section showing the connection (arrowheads) of the pharynx with the body‐wall musculature. (c,h) are lateral views and (a,b,d,e) are dorsal views. Anterior is up in (a–g) and left in (h). *Scale bars*: (a,e,h) 50 μm; (b,c) 25 μm and (d) 10 μm. br, brain; cm, circular muscle fibers; ey, eyes; gv, granular vesicle; lm, longitudinal muscle fibers; me, muscularis externa; mi, muscularis interna; ogo, orogenital opening; ph, pharynx; phl, pharynx lumen; rm, radial muscle fibers; rsm, ring‐shaped muscles; sv, seminal vesicles

**Figure 4:**
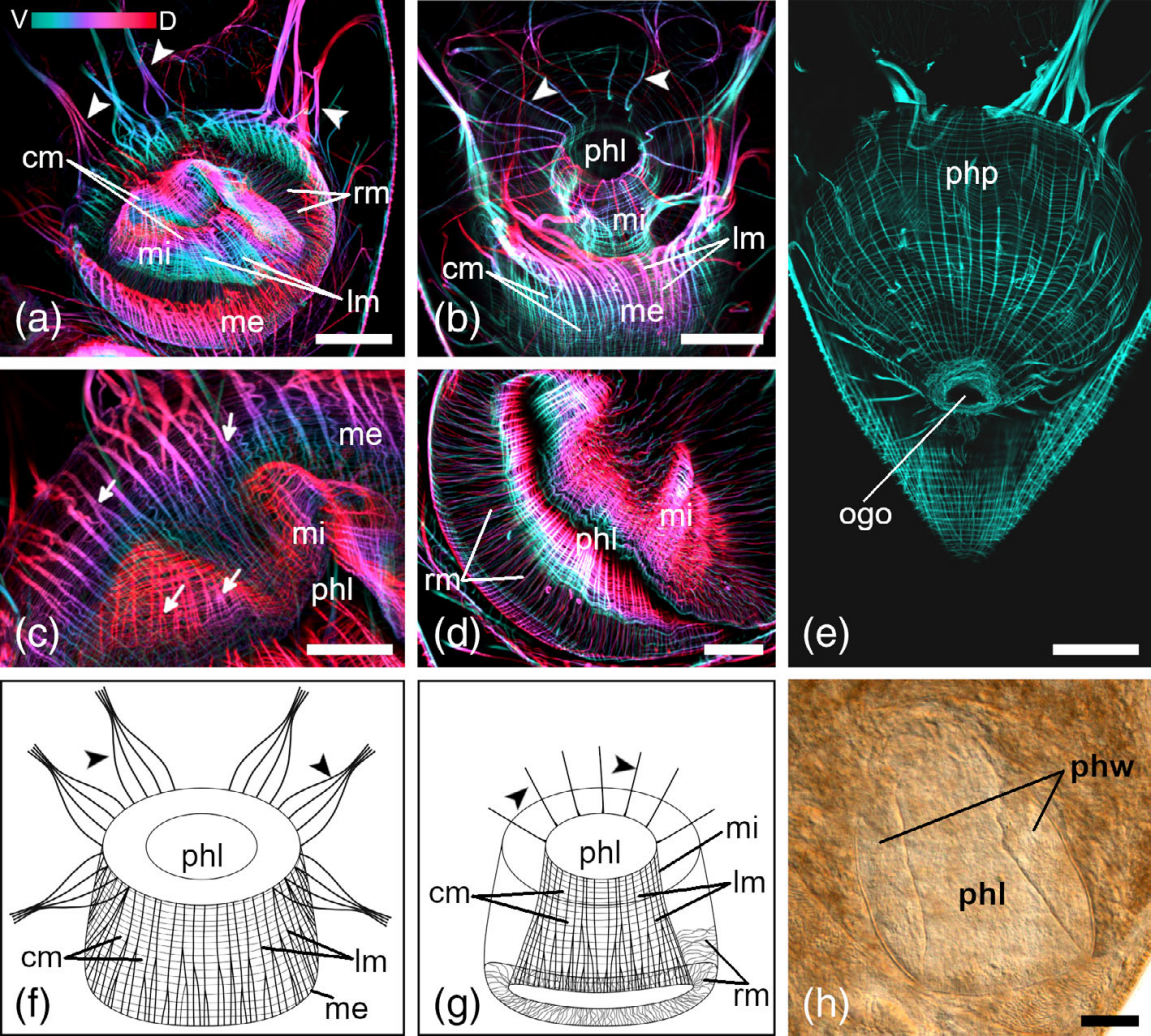
Pharyngeal musculature of *Cylindrostoma monotrochum*. (a–d) Depth‐color‐coded central projections, blue is more ventral, red more dorsal. White structures originate from an accumulation of individual hues from corresponding pixels across each plane of the confocal stack during image processing. (a,b) Overview of the whole pharynx seen from slightly different angles. (c) Close‐up of longitudinal muscle fibers. Arrows show branching points of lm of the muscularis externa and interna. (d) Distal view showing connection between muscularis interna and externa with radial muscle fibers. (e) Overview of the pharyngeal pouch leading to the orogenital opening. Multiple projections of different layers were stitched together and subjected to different brightness and contrast adjustments to allow for a better view of the pouch. (f,g) Schematic drawings of the muscularis externa (f) and interna (g). (h) Differential interference contrast of a squeeze preparation of a live adult specimen. The pharyngeal wall surrounds the conical lumen of the pharynx. Arrowheads in (a,b,f,g) point at the anchoring muscle fibers of the muscularis externa (a,f) and interna (b,g). Anterior is up in all panels. *Scale bars*: (a,b,e,h) 50 μm and (c,d) 25 μm. cm, circular muscle fibers; lm, longitudinal muscle fibers; me, muscularis externa; mi, muscularis interna; ogo, orogenital opening; phl, pharynx lumen; php, pharyngeal pouch; phw, pharyngeal wall; rm, radial muscle fibers

The musculature of the pharynx is prominent in stack projections of mature animals (Figures 3 and 4). The pharynx wall is composed of a muscularis externa (me) and a muscularis interna (mi; Figures 3a,b,f,g and 4a,b,f,g). The me is built of outer, thick lm (2–4 μm) and of inner, thin cm (0.6 μm). While lm are well spaced out, cm are directly adjacent to each other (Figures 3a,f and 4a,b,f). The mi has the same characteristics as the me (Figures 3b,g and 4b,g), except that lm are thinner (0.8–1.5 μm) and toward the distal end they begin to branch (Figures 3d,g and 4c,f,g). The cm of the mi are too thin and too close together to measure them accurately. From the middle to the distal end of the pharynx, irregular radial muscles connect the me with the mi (Figures 3d,g and 4a,d,g). An unusual pattern of lm radiates out from the proximal end of the me, forming bundles at the periphery of the pharynx (Figures 3a,c,e,f and 4a,f). The bundles are reminiscent of the shape of the petals of a crocus blossom. Histological sections confirmed that these bundles build a connection between the pharynx and the body‐wall musculature (Figure 3c,h). From the mi also some muscle fibers run toward the body‐wall and build a connection to the body‐wall musculature (Figures 3b,g and 4b,g). However, it was not clearly visible if the bundles attach to a specific layer of the body‐wall musculature. A spider net‐like structure of thin lm and cm surround the pharynx and form the pharyngeal pouch (Figure 4e). Posterior to the pharynx, the pouch gets narrower and leads straight to the orogenital opening. A sphincter muscle surrounds the orogenital opening (Figures 1e, 2e, 3e, and 4e).

#### 3.2.2 Differences between pharyngeal musculature of *M. striatum* and *C. monotrochum*

The pharynx of *M. striatum* is smaller in size even though the muscle fibers are thicker, and they have a more regular pattern than in *C. monotrochum* (Figures 3 and 4). The diameter of the pharynx of *M. striatum* is about 103 ± 5.4 μm (*n* = 10), whereas the diameter of the pharynx of *C. monotrochum* is about 150 ± 6.4 μm (*n* = 4). The shape of the pharyngeal lumen is also different, cylindrical in *M. striatum* and conical in *C. monotrochum* (Figures 3b,g and 4a,g,h). The lm also show some differences between the two species. In *C. monotrochum*, but not in *M. striatum*, the lm not only of the mi, but also of the me begin to branch toward the distal end (Figures 3d,f,g and 4c,f,g). In *M. striatum*, interspaces between lm of the mi are four times wider than of the me (Figure 3a,b,f,g), while in *C. monotrochum* lm of the me are only one and a half times wider than of the mi (Figure 4a,b,f,g). The petal‐shaped (or anchoring) muscles of *M. striatum* are built of 10, evenly distributed bundles, which are formed of five to seven strong lm (Figure 3a,c,f). From the mi about 10 muscle fibers with a thickness of 3 μm run toward the body‐wall (Figure 3b,g). In *C. monotrochum*, the petal‐shaped muscles are built of eight, unevenly distributed muscle bundles (Figure 4a,f). The eight bundles are formed of four strong muscle fibers, which themselves are built of two or three lm from the me. About 15 muscle fibers with a thickness of 1.5 μm radiate out from the mi toward the body‐wall (Figure 4b,g).

#### 3.2.3 General characteristics of the musculature of the genital organs

**Figure 5:**
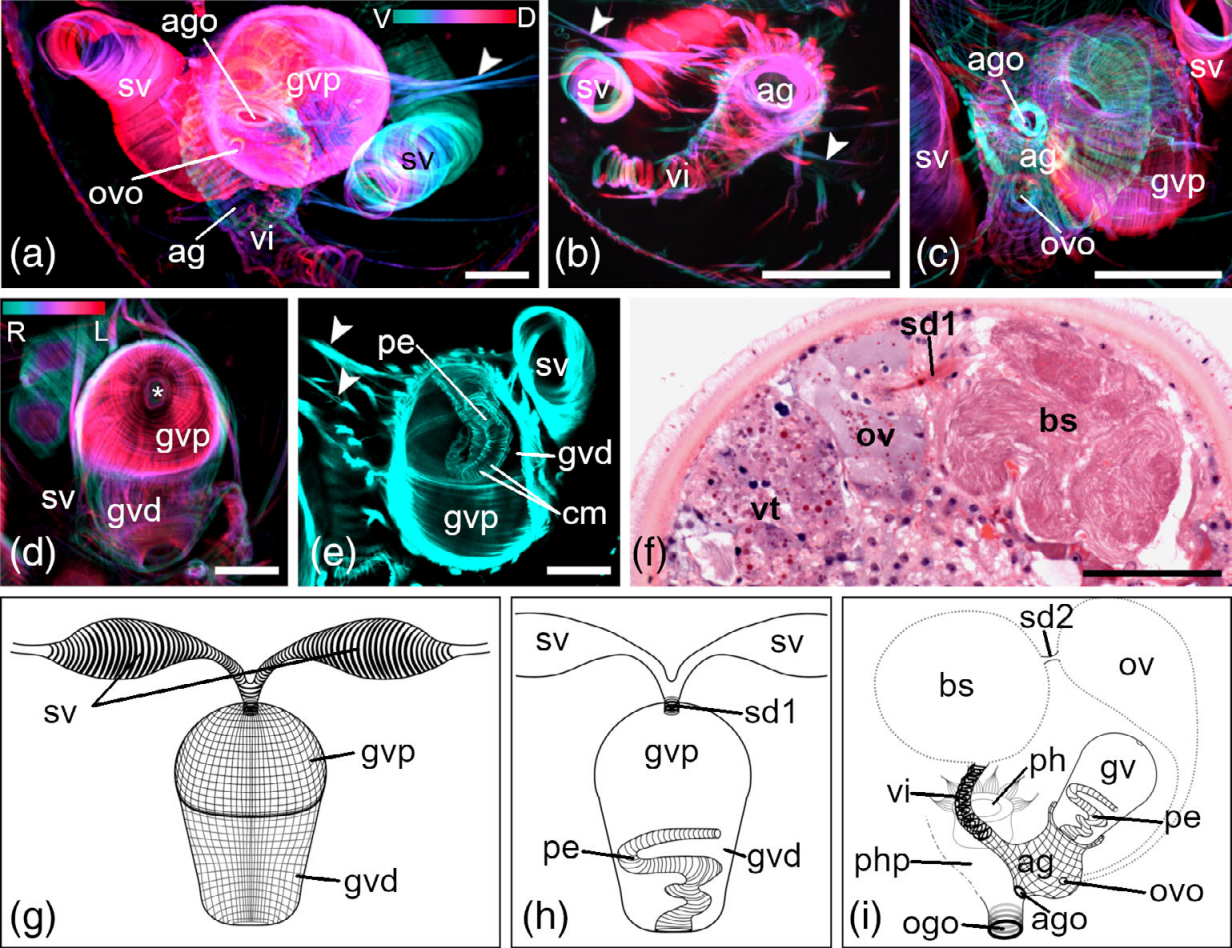
Musculature of genital organs of *Monoophorum striatum*. (a–d) Depth‐color‐coded central projections, in (a–c) blue is more ventral, red more dorsal, in (d) blue is more right, red more left. White structures originate from an accumulation of individual hues from corresponding pixels across each plane of the confocal stack during image processing. (a) Overview of both seminal vesicles connected to the granular vesicle; the latter and the vagina interna are entering into the atrium genitale, which opens into the pharyngeal pouch. (b) Detail of vagina interna entering the atrium genitale. (c) Detail of the atrium genitale. (d) Detail of the granular vesicle, asterisk marks the opening of the seminal duct. (e) Central projection showing the long penis invaginated inside the granular vesicle. Arrowheads in (b,e) point at muscle fibers connecting the atrium genitale with the body‐wall. (f) Histological section showing the connection (sperm duct) between ovary and bursa seminalis. (g–i) Schematic drawings of (g,h) seminal and granular vesicles, and the penis; (i) of the whole genital apparatus; the bursa seminalis is connected to the atrium genitale via the vagina interna. (a–c,e,g–i) Dorsal views, anterior is up, (d,f) lateral views, anterior is left, dorsal is up. *Scale bars*: (a–e) 25 μm and (f) 50 μm. ag, atrium genitale; ago, opening of the atrium genitale into the pharyngeal pouch; bs, bursa seminalis; cm, circular musculature; gvd, distal part of the granular vesicle; gvp, proximal part of the granular vesicle; ogo, orogenital opening; ov, ovary; ovo, opening of the oviduct into the atrium genitale; pe, penis; ph, pharynx; php, pharyngeal pouch; sd1, seminal duct connecting seminal vesicles sv with the gv; sd2, seminal duct connecting bs and ov; vi, vagina interna; vt, vitellaries

**Figure 6:**
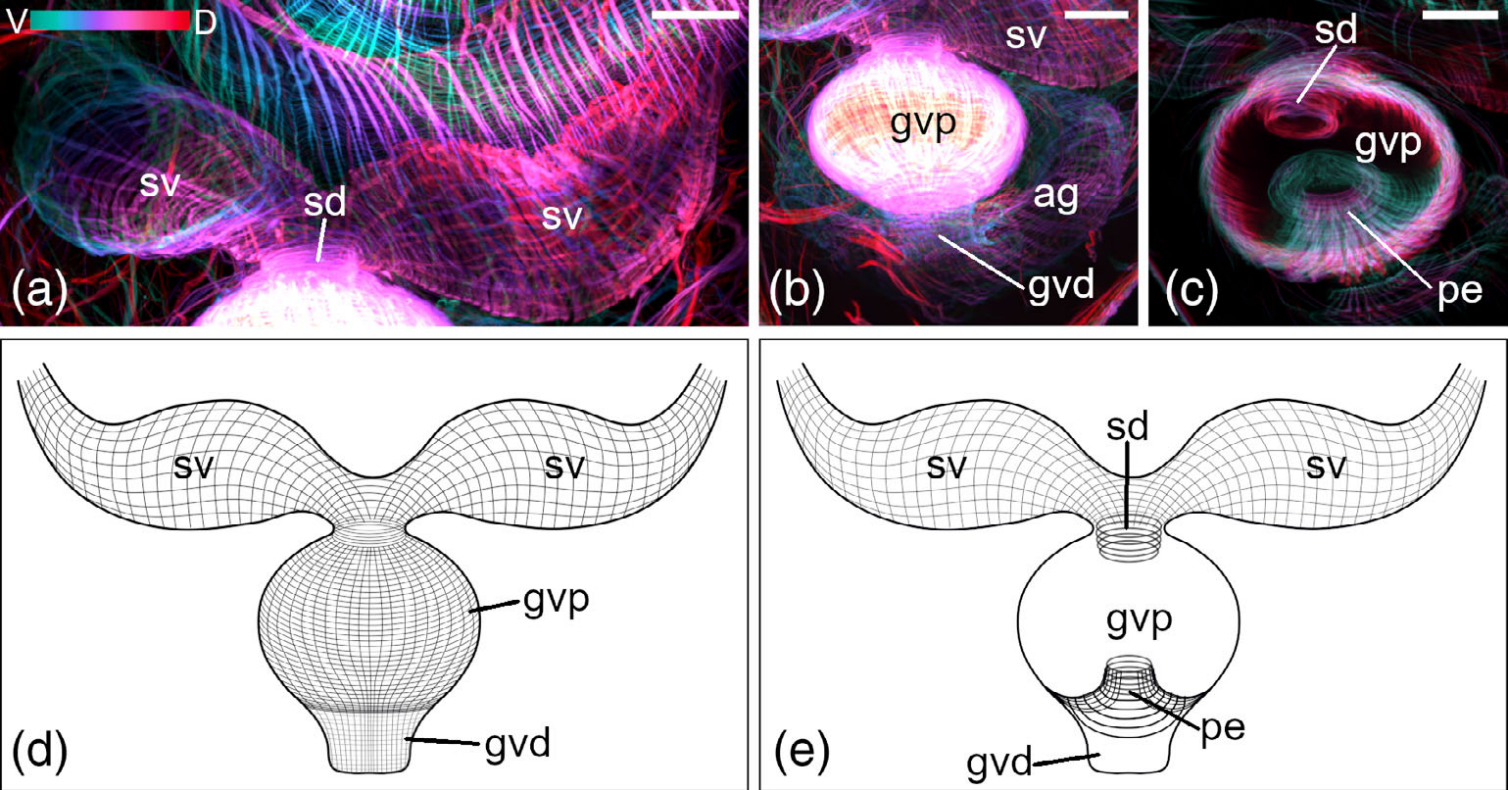
Musculature of the male genital organ of *Cylindrostoma monotrochum*. (a‐c) Depth‐color‐coded central projections, blue is more ventral, red more dorsal. White structures originate from an accumulation of individual hues from corresponding pixels across each plane of the confocal stack during image processing. (d,e) Schematic drawings of the external (d) and the internal view (e) of the granular vesicle gv. (a,d,e) The paired seminal vesicles are located on either side of the gv. (b,d) Fine longitudinal and circular muscle fibers build the wall of the gv. (b) The gv opens into the atrium genitale. (c,e) Inside the gv fine circular muscle fibers line the seminal duct, and circular and longitudinal muscle fibers build the penis. All panels are dorsal views and anterior is up. *Scale bars*: 25 μm. ag, atrium genitale; gvd, distal part of the granular vesicle; gvp, proximal part of the gv; pe, penis; sd, seminal duct; sv, seminal vesicles

The musculature of male genital organs is also prominent in stack projections of mature animals (Figures 5 and 6). The granular vesicle is most noticeable (Figures 5a,d–g and 6b,d). It can be divided in a proximal and a distal part (Figures 5d,g and 6b,d). The musculature of the wall of the granular vesicle, consisting of thin lm and cm, is denser in the proximal part than in the distal part. Not all lm from the proximal part are continuous with the lm from the distal part, but they stop at the end of the proximal part of the granular vesicle (Figures 5d,g and 6b,d). The lm constitute the outer layer and the cm the inner layer. At the distal end, the granular vesicle opens into a funnel‐shaped atrium genitale (Figure 5c,i). A net formed of thicker lm and thinner cm surrounds the atrium genitale. At the proximal end of the granular vesicle, a small sphincter muscle encircles the opening of the seminal duct (Figure 5d,h). The musculature of the seminal duct is continuous with the musculature of the paired seminal vesicles (Figures 5g and 6a,d). Several muscle fibers connect the granular vesicle and the atrium genitale to the body‐wall musculature (Figure 5a,b,e), but it was not clearly visible to which body‐wall muscle layer.

#### 3.2.4 Differences between the musculature of the genital organs of *M. striatum* and *C. monotrochum*

In *M. striatum*, the musculature of the paired seminal vesicles consists only of thick cm (4 μm; Figure 5a,g), while in *C. monotrochum*, it consists of a loose grid of thin cm (0.8–1 μm) and lm (1–1.5 μm; Figure 6a,d). A peculiarity of *M. striatum* is its long penis. At the resting state, the penis is invaginated into the granular vesicle (Figure 5e,h). A fine structure of thin cm builds the musculature of the penis. No lm muscles can be seen. In *C. monotrochum*, the penis is rather short and the musculature consists of cm and lm originating from the musculature of the granular vesicle. Inside the atrium genitale of *M. striatum* are two sphincter muscles (Figure 5c,i). One of the sphincters surrounds the opening into the pharyngeal pouch. The second sphincter muscle surrounds the opening of the oviduct into the atrium genitale, but no musculature of the duct can be seen. The musculature of the vagina interna is formed of cm and leads from the atrium genitale into the nonmuscled bursa seminalis (Figure 5b,i). The bursa seminalis is connected to the ovaries through a seminal duct (Figure 5f). In the atrium genitale of *C. monotrochum* no sphincter muscles are seen, but one individual shows a canal, which runs from the anterior part of the body along the pharynx to the atrium genitale. According to the histological sections, this canal is the oviduct (data not shown).

#### 3.2.5 General characteristics of additional musculature

**Figure 7:**
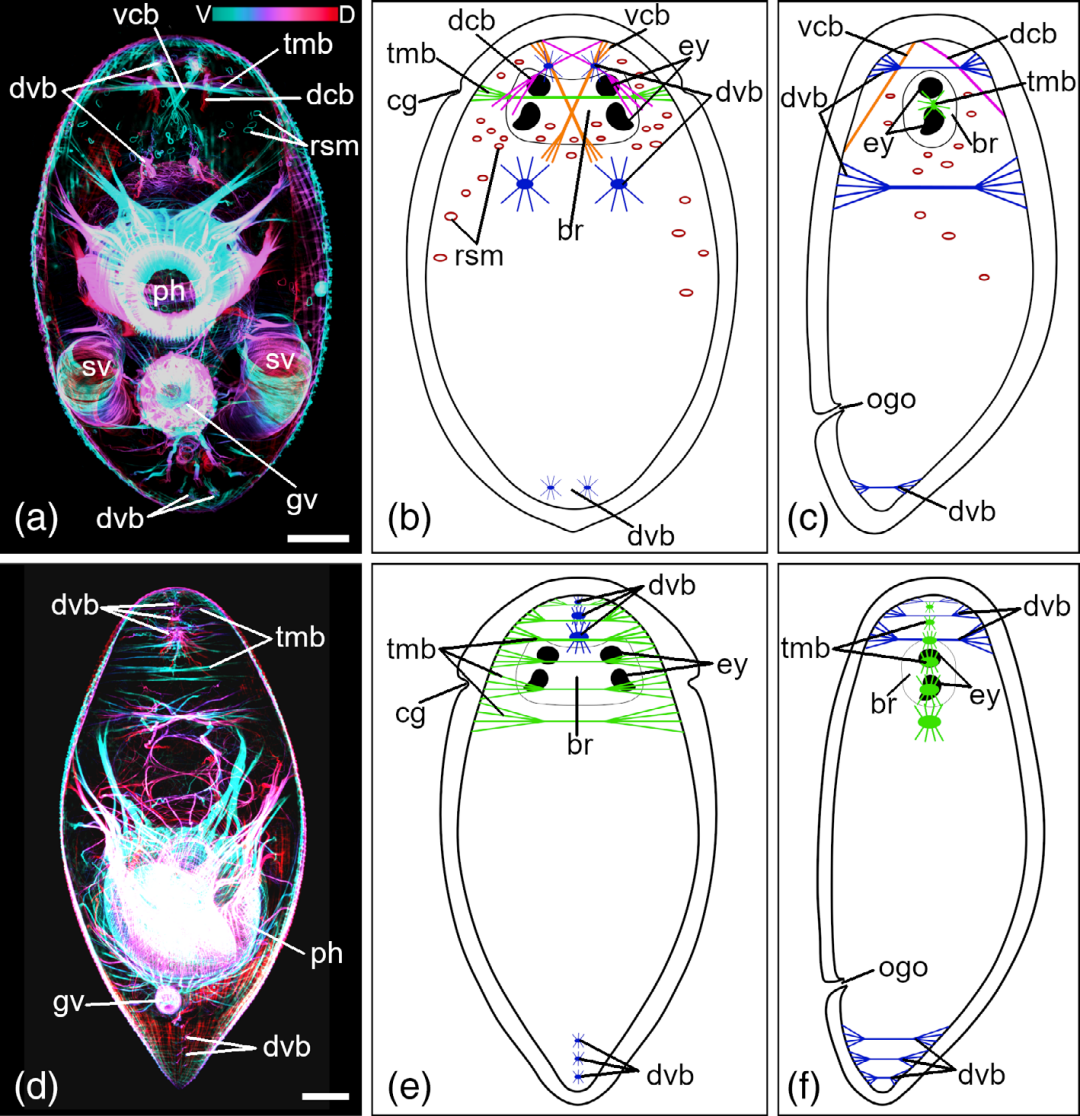
Additional musculature of *Monoophorum striatum* and *Cylindrostoma monotrochum*. (a,d) Depth‐color‐coded central projections, blue is more ventral, red more dorsal. White structures originate from an accumulation of individual hues from corresponding pixels across each plane of the confocal stack during image processing. (a–c) *M. striatum*. (d–f) *C. monotrochum*. (b,c,e,f) Schematic drawings of additional musculature. (a,b,d,e) Dorsal views, (c,f) lateral views, taken from lateral views of some individuals. Anterior is up in all panels. *Scale bars*: 50 μm. br, brain; cg, ciliary groove; dcb, dorsal crossover bundles; dvb, dorsoventral muscle bundles; ey, eyes; gv, granular vesicle; ogo, orogenital opening; ph, pharynx; rsm, ring‐shaped muscles; sv, seminal vesicle; tmb, transverse muscle bundles; vcb, ventral crossover bundles

In both species, dorsoventral muscle bundles (dvb), which connect the dorsal and the ventral sides of the body, are present only in the anterior and the posterior parts of the body and in low numbers (Figure 7). Each consists of several muscle fibers forming a bundle, and at both ends they fan out. There are also transverse muscle bundles (tmb) in the anterior part of the body which build connections between the left and the right sides of the body (Figure 7). Like the dvb, each consists of several muscle fibers, and they begin to fan out on both sides near the body‐wall. Posterior to the brain, some tmb follow the ciliary groove (Figure 7a,b,d,e). There were no muscle fibers traversing or encapsulating the brain.

#### 3.2.6 Differences between additional musculature of *M. striatum* and *C. monotrochum*

Tmb and dvb are more pronounced in *C. monotrochum* than in *M. striatum*. *C. monotrochum* has six to eight tmb in the anterior fourth of the body and three dvb in the area posterior to the brain (Figure 7d–f). *M. striatum* only has one tmb at the area of the ciliary groove and one pair of dvb anterior and one pair posterior to the brain (Figure 7a–c). At the posterior end, *C. monotrochum* has at least three dvb, whereas in *M. striatum* only one pair of dvb can be seen (Figure 7a,b). *M. striatum* shows some peculiarities that are not found in *C. monotrochum*. A pair of ventral and a pair of dorsal crossover muscle bundles run from the anterior tip of the body toward the ventral and the dorsal body‐wall musculature, respectively (Figure 7a–c). The muscle bundles of the dorsal pair cross each other in front of the brain, whereas the muscle bundles of the ventral pair cross each other at the level of the eyes. Unusual ring‐shaped muscles (rsm) are distributed all over the body. Most of them are in the front of the body in the area of the testis follicles (Figure 7a), but they could not be correlated to any structure. However, rsm are only present in 10 animals out of 20. In *C. monotrochum*, no rsm can be seen.

## 4 DISCUSSION

### 4.1 Body‐wall musculature

*M. striatum* and *C. monotrochum* have a typical rhabditophoran body‐wall musculature consisting of an outer layer of circular muscle rings, a layer of two different types of diagonal muscle fibers, and an inner layer of longitudinal muscle fibers. In contrast, Böhmig (1890) described the body‐wall musculature of *M. striatum* as weak with only two layers, an outer layer of thin circular muscle fibers and an inner layer of thin longitudinal muscle fibers. Ritter‐Záhony (1908) also reported only two different layers, an outer circular and an inner longitudinal layer, in the body‐wall musculature of *C. monotrochum*. Neither Böhmig, nor Ritter‐Záhony mentioned any diagonal muscle fibers in the body‐wall of *M. striatum* and *C. monotrochum*. The fact that Böhmig and Ritter‐Záhony did their observations on histological sections might explain that they overlooked diagonal muscle fibers in their studies.

Böhmig (1890) reported a single set of diagonal muscle fibers only in three plagiostomids, *Vorticeros auriculatum* (Müller, 1784), *Plagiostomum chromogastrum* von Graff, 1904, and *Plagiostomum sulphureum* von Graff, 1882. Karling (1940) investigated also three plagiostomid species, *Acmostomum dioicum* Metschnikoff, 1865, *Plagiostomum torquatum* Karling, 1940, and *Plagiostomum norvegicum* (Karling, 1940). While he found no diagonal muscles in the first two species, he described a single set of diagonal muscles in *P. norvegicum*. So far, two different types of diagonal muscle fibers have only been described for members of the family Pseudostomidae.

Furthermore, Böhmig (1890) described an additional layer of thick longitudinal muscle fibers on the ventral side and the lateral body parts of *M. striatum*. Our results confirm these observations, but only for the ventral side. On the lateral body parts, we did not find additional longitudinal fibers. Karling (1940) stated that all investigated pseudostomids (*Pseudostomum klostermanni* (von Graff, 1874), *Pseudostomum quadrioculatum* (Leuckart, 1847), and *Allostoma durum* (Fuhrmann, 1896)) show a creeping sole‐like reinforcement of the longitudinal muscle fibers on the ventral side. We can confirm this only for *M. striatum*.

It has been suggested that the diagonal muscle fibers of the body‐wall derive from either longitudinal or circular muscle fibers (Rieger et al., 1991). An immunohistochemical study on *Dugesia japonica* Ichikawa & Kawakatsu, 1964 showed that the circular muscle rings of the body‐wall are built of myosin heavy chain A (MHC‐A), while longitudinal and diagonal muscle fibers are built of MHC‐B. These findings suggest that diagonal muscle fibers derive from longitudinal muscle fibers (Orii et al., 2002). Our own findings partially support this notion based on the orientation of the diagonal muscles type 1, which are likely derived from longitudinal muscles. On the other hand, the orientation of the diagonal muscles type 2 and crossover diagonal muscles makes them unlikely to originate from longitudinal muscles, but rather derive from circular muscles. To address this issue, we tried antibodies made against triclad muscle types, specifically for Smed 6G10 (circular and diagonal) and 2G3 (longitudinal, diagonal, and circular; Ross et al., 2015)—however, the stainings did not work (data not shown).

### 4.2 Pharyngeal musculature

In contrast to the pharynx plicatus, the pharynx bulbosus shows a septum of musculature and extracellular matrix that closes the connection with the parenchyma, and it has a reduced pharyngeal pouch (Ehlers, 1985; von Graff, 1882; Rieger et al., 1991). *M. striatum* and *C. monotrochum* do not show any sign of a septum separating the pharynx and the parenchyma. Additionally, the pharyngeal pouch of both species enwraps the whole pharynx. Thus, we confirm statements of Ritter‐Záhony (1908) and Karling (1940) that both species have a pharynx of the type plicatus, just like most other pseudostomids.

Our results show an additional character of the pseudostomid pharynx. Strong longitudinal muscle fibers radiating out of the pharynx formed a petal‐like structure, and built the connection with the body‐wall musculature. Probably, these muscle bundles play the role of anchoring muscles. In an older work based on histological sections, such muscles were already described in *C. monotrochum*, but were referred to as retractor muscles (Ritter‐Záhony, 1908). Regardless of the name, these muscles are reminiscent of the anchoring muscles in the triclads *Girardia tigrina* (Girard, 1850) and *Polycelis tenuis* Ijimo, 1884, linking the proximal part of the pharynx with the body‐wall (Kreshchenko, 2017; Kreshchenko et al., 1999). However, the triclad anchoring muscles consist of many, relatively thin fibers, compared to the thick and few bundles in the prolecithophorans.

In the fecampiid *Urastoma cyprinae* (von Graff, 1882), previously classified as a prolecithophoran, few muscles, which radiate out from the proximal end and the middle of the pharynx, were hypothesized to connect the pharynx and gut (Hooge & Tyler, 1999). These muscles are reminiscent of the prolecithophoran anchoring muscles, as there are only few of them evenly distributed on the base and in the middle of the pharynx.

In the rhabdocoel *Castrella* Fuhrmann, 1900, no anchoring muscles have been described, but so‐called protractor muscles attach at the distal tip of the pharynx and run toward the proximal base and further extend to the body‐wall (Kotikova, Raikova, Reuter, & Gustafsson, 2002). However, the shape and path of these protractors is dissimilar to the anchoring muscles of prolecithophorans, triclads, and fecampiids. Lastly, in the proseriate *Monocelis* Ehrenberg, 1831 the proximal base of the pharynx is shown to be also attached to the body‐wall in a way similar to that seen in triclads, but the fibers are much less numerous and wider apart in the proseriate (Girstmair et al., 2014).

### 4.3 Musculature of the genital organs

Characters of the genital organs are commonly used for species determination in prolecithophorans and also in many other free‐living flatworms (Böhmig, 1890; Doe, 1982; Ladurner, Mair, Reiter, Salvenmoser, & Rieger, 1997; Luther, 1960; Rieger, 1977). In prolecithophorans, the male part usually consists of a copulatory organ (penis), a granular vesicle, and one or two seminal vesicles, while the female genital apparatus is composed of a vagina (interna and/or externa) and a bursa seminalis (Westblad, 1955). Böhmig (1890) and Karling (1940) already stated that the granular vesicle in pseudostomids and plagiostomids is quite muscular, which we can confirm with our results. Overall, our phalloidin stainings corroborate the findings of Westblad (1955) in that a long penis is invaginated into the granular vesicle in *M. striatum*. Here, we show that the musculature of the penis only consists of circular muscle fibers (Figure 5e,h,i), while that of *C. monotrochum* features both circular and longitudinal muscles (Figure 6c,e), a condition also found in many other flatworms as well as acoels (Egger et al., 2009; Girstmair et al., 2014; Zauchner, Salvenmoser, & Egger, 2015). Intriguingly, also the seminal vesicles of *M. striatum* lack longitudinal muscle fibers. In the species description of *C. monotrochum*, the seminal vesicles are described as “false” seminal vesicles due to the apparent lack of musculature (Ritter‐Záhony, 1908), while we can unambiguously show that the seminal vesicles are lined with musculature (Figure 6a,d,e).

### 4.4 Additional musculature

Most free‐living flatworms have poorly developed dorsoventral musculature limited to specific body parts, usually in the head or in the tail (Ehlers, 1985; Rieger et al., 1991). *Catenula lemnae* Dugès, 1832 shows almost no dorsoventral musculature and *Macrostomum hystricinum marinum* Rieger, 1977 shows only few dorsoventral muscle fibers in the rostrum and in the region of the adhesive plate (Ehlers, 1985; Rieger et al., 1994). Larger flatworms show more developed dorsoventral musculature (Rieger et al., 1991). In *Monocelis* sp. and *D. japonica* dorsoventral musculature is present throughout the whole body (Girstmair et al., 2014; Orii et al., 2002). *D. japonica* shows additional transverse muscle fibers which either cross through the body or connect the edges with the ventral body‐wall or dorsoventral muscle fibers (Orii et al., 2002). Böhmig (1890) reports scarce dorsoventral and sagittal muscles in *V. auriculatum* and some other *Plagiostomum* Schmidt, 1852 species, and these are restricted to the front and back end of the body. Our results agree with Böhmig's observations. *M. striatum* and *C. monotrochum* showed few muscle fibers connecting the dorsal and the ventral body‐wall as well as muscle fibers running transversely through the body. In the fecampiid *U. cyprinae*, neither transverse, nor dorsoventral muscle fibers were shown (Hooge & Tyler, 1999)—this is in stark contrast to triclads, which have transverse and dorsoventral musculature over the whole body (Kreshchenko, 2017; Orii et al., 2002).

The rsm found in half of the phalloidin‐stained specimens of *M. striatum* (collected in October) are possibly associated with their life cycle. In March and in October, we found specimens with a copulatory organ and with testis follicles. In May, specimens had a copulatory organ, but we could not unambiguously identify testis follicles, and the phalloidin staining of these animals did not show any rsm. Specimens from March were only used for histology and not for phalloidin stainings, so we do not know about rsm in animals from March. To clarify the connection of rsm with the life cycle, additional observations about the presence of absence of testis follicles in May, and of rsm in March are needed.

In specimens of *C. monotrochum* collected in October, we observed animals with no copulatory organ and no testis follicles, but also animals with copulatory organ, but without testis follicles, and animals with both, copulatory organ and testis follicles. None of the phalloidin‐stained animals showed rsm.

Ring‐shaped muscles were not previously described in works based on histological sections (Böhmig, 1890; von Graff, 1913; Westblad, 1955). A structural analysis by transmission electron microscopy can help to further investigate the nature of the rsm.

### 4.5 Systematic considerations

In molecular phylogenies, the genus *Cylindrostoma* Ørsted, 1845 has been recovered as a member of Pseudostomidae, but *Cylindrostoma*, as well as *Pseudostomum*, are not monophyletic genera (Norén & Jondelius, 1999, 2002). *Monoophorum* Böhmig, 1890 has not been included in any molecular phylogeny yet, but shared morphological characters with *Cylindrostoma* and *Pseudostomum*, such as a ciliary groove at the level of the brain, an encapsulated brain with two pairs of eyes, and an orogenital opening on the ventral side at the posterior body half make *Monoophorum* a likely pseudostomid. In terms of the musculature, *M. striatum* and *C. monotrochum* show many shared characters. Both species have the same pattern of body‐wall musculature, namely, showing circular, longitudinal, and two different types of diagonal muscles. Thick muscle bundles, which are extensions of the longitudinal muscles from the muscularis externa and interna of the pharynx, build the anchoring muscles of the pharynx. Still, we find some idiosyncrasies in the musculature differentiating the two observed species. While *M*. *striatum* has a creeping sole‐like strengthening of the longitudinal muscles of the body‐wall on the ventral side, *C. monotrochum* does not. The musculature of the seminal vesicles in *M. striatum* consists only of circular muscles and in *C. monotrochum* it has both circular and longitudinal muscles. The musculature of the penis in *M. striatum* consists only of circular muscles, whereas that in *C. monotrochum* has both circular and longitudinal muscles. *M. striatum* has fewer dorsoventral and transverse muscles than *C. monotrochum*, but *M. striatum* has special inner crossover muscles at the level of the brain. We could not find any common character for the adiaphanidan clade, which consists of the Prolecithophora, the Fecampiida Rohde, Luton, Baverstock, & Johnson, 1994 and the Tricladida Lang, 1884. The pattern of the body‐wall musculature of all three taxa shows the general rhabditophoran pattern as they consist of an outer circular muscle layer, an inner longitudinal muscle layer, and a diagonal muscle layer in between. All three taxa show anchoring muscles of the pharynx, but the numerous and thin anchoring muscles of the triclads differ from the few and thick anchoring muscles of the prolecithophorans and the fecampiids.

## CONFLICT OF INTEREST

The authors declare no potential conflict of interest.

## AUTHOR CONTRIBUTIONS

A.L.G. and B.E. designed the study. A.L.G. performed stainings, and A.L.G. and P.B. made confocal stacks and prepared the figures. A.L.G. and B.E. wrote the manuscript. All authors agreed on the final manuscript.

## References

[jmor21039-bib-0001] Böhmig, L. (1890). Untersuchungen über rhabdocöle Turbellarien. II. Plagiostomina und Cylindrostomina Graff. Zeitschrift für Wissenschaftliche Zoologie, 51, 167–479.

[jmor21039-bib-0002] Doe, D. A. (1982). Ultrastructure of copulatory organs in Turbellaria. Zoomorphology, 101(1), 39–59. 10.1007/BF00312029

[jmor21039-bib-0003] Dugès, A. (1832). Description d'un zoophyte, voisin des Bothriocephales (*Catenula lemnae* Nob). Annales des sciences naturelles, 1(26), 198–205.

[jmor21039-bib-0004] Egger, B. , Gschwentner, R. , Hess, M. W. , Nimeth, K. T. , Adamski, Z. , Willems, M. , … Salvenmoser, W. (2009). The caudal regeneration blastema is an accumulation of rapidly proliferating stem cells in the flatworm *Macrostomum lignano* . BMC Developmental Biology, 9(1), 1–15. 10.1186/1471-213X-9-41 19604404 PMC2717932

[jmor21039-bib-0005] Ehlers, U. (1985). Das Phylogenetische System der Platyhelminthes. Stuttgart and New York: Gustav Fischer.

[jmor21039-bib-0006] Ehrenberg, C. G. (1831). Animalia evertebrata. Pars Zoologica: Symbolae physicae seu icones et descriptiones naturalium novorum aut minus cognitorum quae ex itineribus Lybiam Aegyptum Nubiam Dongalam Syriam Arabiam et Habessinian.

[jmor21039-bib-0007] Fuhrmann, O. (1896). Note faunique sur les Turbellaries rhabdocoeles de la Baie de Concarneau. *Comptes rendus des séances de la Société de la biologie et de ses filiales* . Tom, 48(10. sér. T. 3), 1011–1013.

[jmor21039-bib-0008] Fuhrmann, O. (1900). Note sur les Turbellaries des environs de Geneve. Revue Suisse de Zoologie, 7, 717–731.

[jmor21039-bib-0009] Girard, C. (1850). A brief account of the fresh water species of planarian inhabitationg the United States. Proceedings of the Boston Society of Natural History, 3, 264–265.

[jmor21039-bib-0010] Girstmair, J. , Schnegg, R. , Telford, M. J. , & Egger, B. (2014). Cellular dynamics during regeneration of the flatworm *Monocelis* sp. (Proseriata, Platyhelminthes). EvoDevo, 5(1), 37 10.1186/2041-9139-5-37 25908954 PMC4407785

[jmor21039-bib-0011] von Graff, L. (1874). Zur Kenntniss der Turbellarien. Zeitschrift für Wissenschaftliche Zoologie, 24, 123–160.

[jmor21039-bib-0012] von Graff, L. (1878). Kurze Berichte über fortgesetzte Turbellarienstudien 1. Zeitschrift für Wissenschaftliche Zoologie, 30, 457–465.

[jmor21039-bib-0013] von Graff, L. (1882). Monographie der Turbellarien I. Leipzig: Rhabdocoelida.

[jmor21039-bib-0014] von Graff, L. (1904–08). Acoela und Rhabdocoela. Plathelminthes III. Turbellaria In Bronn's Klassen und Ordnungen des Tierreichs Vol. IV, Abt. 1c. Leipzig: Winter'sche Verlagsbuchhandl.

[jmor21039-bib-0015] von Graff, L. (1911). Acoela, Rhabdocoela und Allococoela des Ostens der Vereinigten Staaten von Amerika. Zeitschrift für Wissenschaftliche Zoologie, 99, 1–108.

[jmor21039-bib-0016] von Graff, L. (1913). Turbellaria II. Rhabdocoelida In Das Tierreich. Eine Zusammenstellung und Kennzeichnung der rezenten Tierformen 35. Berlin: Verlag von R. Friedländer und Sohn.

[jmor21039-bib-0017] Hooge, M. D. (2001). Evolution of body‐wall musculature in the Platyhelminthes (Acoelomorpha, Catenulida, Rhabditophora). Journal of Morphology, 249(3), 171–194. 10.1002/jmor.1048 11517463

[jmor21039-bib-0018] Hooge, M. D. , & Tyler, S. (1999). Musculature of the facultative parasite *Urastoma cyprinae* (Platyhelminthes). Journal of Morphology, 241(3), 207–216. 10461131 10.1002/(SICI)1097-4687(199909)241:3<207::AID-JMOR3>3.0.CO;2-S

[jmor21039-bib-0019] Ichikawa, A. , & Kawakatsu, M. (1964). A new freshwater planarian, *Dugesia japonica*, commonly but erroneously known as *Dugesia gonocephala* . Annotationes Zoologicae Japonenses, 37(3), 185–194.

[jmor21039-bib-0020] Ijimo, J. (1884). Untersuchungen über den Bau und die Entwicklungsgeschichte der Süsswasserdendrocölen (Tricladen). Zeitschrift für Wissenschaftliche Zoologie, 40, 359–454.

[jmor21039-bib-0021] Jondelius, U. , Norén, M. , & Hendelberg, J. (2001). The Prolecithophora In Littlewood D. T. J. & Bray R. A. (Eds.), Interrelationships of the Platyhelminthes (pp. 74–80). London: Taylor .

[jmor21039-bib-0022] Karling, T. G. (1940). Zur Morphologie und Systematik der Alloeocoela Cumulata und Rhabdocoela Lecithophora (Turbellaria). Acta Zoologica Fennica, 26, 1–260.

[jmor21039-bib-0023] Karling, T. G. (1962). Marine Turbellaria from the Pacific coast of North America. II. Pseudostomidae and Cylindrostomidae. Arkiv för Zoologi, 15, 181–209.

[jmor21039-bib-0024] Kotikova, E. , Raikova, O. , Reuter, M. , & Gustafsson, M. K. (2002). The nervous and muscular systems in the free‐living flatworm *Castrella truncata* (Rhabdocoela): An immunocytochemical and phalloidin fluorescence study. Tissue and Cell, 34(5), 365–374. 10.1016/S004081660200037X 12270263

[jmor21039-bib-0025] Kreshchenko, N. (2017). Some details on the morphological structure of planarian musculature identified by fluorescent and confocal laser‐scanning microscopy. Biophysics, 62(2), 271–277. 10.1134/S0006350917020117

[jmor21039-bib-0026] Kreshchenko, N. , Reuter, M. , Sheiman, I. M. , Halton, D. W. , Johnston, R. N. , Shaw, C. , & Gustafsson, M. K. S. (1999). Relationship between musculature and nervous system in the regenerating pharynx in *Girardia tigrina* (Plathelminthes). Invertebrate Reproduction & Development, 35(2), 109–125. 10.1080/07924259.1999.9652375

[jmor21039-bib-0027] Ladurner, P. , Mair, G. R. , Reiter, D. , Salvenmoser, W. , & Rieger, R. M. (1997). Serotonergic nervous system of two Macrostomid species: Recent or ancient divergence? Invertebrate Biology, 116(3), 178–191. 10.2307/3226895

[jmor21039-bib-0028] Lang, A. (1884). Die Polycladen (Seeplanarien) des Golfes von Neapel und der angrenzenden Meeresabschnitte. Eine Monographie In Fauna und Flora des Golfes von Neapel (Vol. 11, pp. 1–688). Leipzig: Wilhelm Engelmann.

[jmor21039-bib-0029] Laumer, C. E. , & Giribet, G. (2017). Phylogenetic relationships within Adiaphanida (Phylum Platyhelminthes) and the status of the crustacean‐parasitic genus *Genostoma* . Invertebrate Biology, 136(2), 184–198. 10.1111/ivb.12169

[jmor21039-bib-0030] Leuckart, R. (1847). Beiträge zur Kenntnis der wirbellosen Thiere. Braunschweig, 4(82–85), 149–150.

[jmor21039-bib-0031] Luther, A. (1960). Die Turbellarien Ostfennoskandiens I. Acoela, Catenulida, Macrostomida, Lecithoepitheliata, Prolecithophora, und Proseriata. Fauna Fennica, 7, 1–155.

[jmor21039-bib-0032] Minot, C. S. (1876). Studien an Turbellarien. Beiträge zur Kenntniss der Plathelminthen. Arbeiten aus dem Zoologisch‐Zootomischen Institut in Würzburg, 3, 405–471.

[jmor21039-bib-0033] Metschnikoff, E. (1865). Zur Naturgeschichte der Rhabdocoelen. Archiv für Naturgeschichte, 31, 174–181.

[jmor21039-bib-0034] Müller, O. F. (1784). Zoologia danica seu animalium Daniae et Norvegiae rariorum ac minus notorum historia descriptiones et historia. Weygand, 2, 1–124.

[jmor21039-bib-0035] Norén, M. (2004). Four new Plagiostomidae (Prolecithophora:Platyhelminthes) from Phuket, Thailand, with a re‐evaluation of Torgeidae Jondelius, 1997, and *Paramultipeniata* Kulinich, 1974. Phuket Marine Biological Center Research Bulletin (Thailand), 65, 9–22.

[jmor21039-bib-0036] Norén, M. , & Jondelius, U. (1999). Phylogeny of the Prolecithophora (Platyhelminthes) inferred from 18S rDNA sequences. Cladistics, 15(2), 103–112. 10.1111/j.1096-0031.1999.tb00252.x 34902908

[jmor21039-bib-0037] Norén, M. , & Jondelius, U. (2002). The phylogenetic position of the Prolecithophora (Rhabditophora, ‘Platyhelminthes’). Zoologica Scripta, 31(4), 403–414. 10.1046/j.1463-6409.2002.00082.x

[jmor21039-bib-0038] Ørsted, A.,. S. (1845). Fortegnelse over Dyr, samlede i Christianiafjord ved Drøbak fra 21–24 Juli, 1844. Naturhistorisk Tidsskrift, Kjøbenhavn, Ser, 2(1), 400–427.

[jmor21039-bib-0039] Orii, H. , Ito, H. , & Watanabe, K. (2002). Anatomy of the planarian *Dugesia japonica* I. The muscular system revealed by antisera against myosin heavy chains. Zoological Science, 19(10), 1123–1131. 10.2108/zsj.19.1123 12426474

[jmor21039-bib-0040] Prudhoe, S. (1985). A monograph on polyclad turbellaria. London: British Museum (Natural History).

[jmor21039-bib-0041] Rieger, R. (1977). The relationship of character variability and morphological complexity in copulatory structures of Turbellaria‐Macrostomida and Haplopharyngida In Sterrer W. & Ax P. (Eds.), Mikrofauna Des Meeresbodens 61. The Meiofauna Species in Time and Space (pp. 197–216). Mainz: Akademie der Wissenschaften und der Literatur.

[jmor21039-bib-0042] Rieger, R. , & Salvenmoser, W. (1991). Demonstration of the muscle‐system in whole mounts of *Macrostomum hystricinum* (Turbellaria, Macrostomida). American Zoologist, 31, 25A.

[jmor21039-bib-0043] Rieger, R. , Salvenmoser, W. , Legniti, A. , & Tyler, S. (1994). Phalloidin‐rhodamine preparations of *Macrostomum hystricinum marinum* (Plathelminthes): Morphology and postembryonic development of the musculature. Zoomorphology, 114(3), 133–147. 10.1007/BF00403261

[jmor21039-bib-0044] Rieger, R. , & Sterrer, W. (1975). New spicular skeletons in Turbellaria, and the occurrence of spicules in marine meiofauna. Journal of Zoological Systematics and Evolutionary Research, 13(4), 207–278. 10.1111/j.1439-0469.1975.tb00509.x

[jmor21039-bib-0045] Rieger, R. , Tyler, S. , Smith, J. P. S., III , & Rieger, G. (1991). Platyhelminthes: Turbellaria In Harrison F. W. & Bogitsch B. J. (Eds.), Microscopic anatomy of invertebrates (pp. 7–140). New York: Wiley‐Liss.

[jmor21039-bib-0046] Ritter‐Záhony, R. v. (1908). Beitrag zur Anatomie von *Allostoma monotrochum* Graff. Mitteilungen des naturwissenschaftlichen Vereins für Steiermark, 44, 147–155.

[jmor21039-bib-0047] Rohde, K. , Luton, K. , Baverstock, P. R. , & Johnson, A. M. (1994). The phylogenetic relationships of *Kronborgia* (Platyhelminthes, Fecampiida) based on comparison of 18S ribosomal DNA sequences. International Journal for Parasitology, 24(5), 657–669. 7928067 10.1016/0020-7519(94)90118-x

[jmor21039-bib-0048] Ross, K. G. , Omuro, K. C. , Taylor, M. R. , Munday, R. K. , Hubert, A. , King, R. S. , & Zayas, R. M. (2015). Novel monoclonal antibodies to study tissue regeneration in planarians. BMC Developmental Biology, 15(2), 2 10.1186/s12861-014-0050-9 25604901 PMC4307677

[jmor21039-bib-0049] Schindelin, J. , Arganda‐Carreras, I. , Frise, E. , Kaynig, V. , Longair, M. , Pietzsch, T. , … Cardona, A. (2012). Fiji: An open‐source platform for biological‐image analysis. Nature Methods, 9(7), 676–682. 10.1038/nmeth.2019 22743772 PMC3855844

[jmor21039-bib-0050] Schmidt, O. (1848). Die rhabdocoelen Strudelwürmer (Turbellaria Rhabdocoela) des süssen Wassers. Jena: Friedrich Mauke.

[jmor21039-bib-0051] Tyler, S. , & Hooge, M. (2004). Comparative morphology of the body wall in flatworms (Platyhelminthes). Canadian Journal of Zoology, 82, 194–210.

[jmor21039-bib-0052] Tyler, S. , Hooge, M. , & Bush, L. M. (2006). Turbellarian taxonomic database. Version 1.7. *Turbellarian Taxonomic Database. Version 1.7*.

[jmor21039-bib-0053] Westblad, E. (1955). Marine “Alloeocoels” (Turbellaria) from North Atlantic and the Mediterranean coasts. I. Arkiv för Zoologi, 7(24), 490–529.

[jmor21039-bib-0054] WoRMS Editorial Board . (2019). *World Register of Marine Species (WoRMS)* http://www.marinespecies.org.

[jmor21039-bib-0055] Zauchner, T. , Salvenmoser, W. , & Egger, B. (2015). A cultivable acoel species from the Mediterranean, *Aphanostoma pisae* sp. nov. (Acoela, Acoelomorpha). Zootaxa, 3941(3), 401–413. 10.11646/zootaxa.3941.3.6 25947519

